# Stable Isotope Models Predict Foraging Habitat of Northern Fur Seals (*Callorhinus ursinus*) in Alaska

**DOI:** 10.1371/journal.pone.0127615

**Published:** 2015-06-01

**Authors:** T. K. Zeppelin, D. S. Johnson, C. E. Kuhn, S. J. Iverson, R. R. Ream

**Affiliations:** 1 National Marine Mammal Laboratory (NMML), Alaska Fisheries Science Center (AFSC), National Oceanic and Atmospheric Administration, Seattle, Washington, United States of America; 2 Department of Biology, Dalhousie University, Halifax, Nova Scotia, Canada; Sonoma State University, UNITED STATES

## Abstract

We developed models to predict foraging habitat of adult female northern fur seals (*Callorhinus ursinus*) using stable carbon (δ^13^C) and nitrogen (δ^15^N) isotope values from plasma and red blood cells. Binomial generalized linear mixed models were developed using blood isotope samples collected from 35 adult female fur seals on three breeding colonies in Alaska during July-October 2006. Satellite location and dive data were used to define habitat use in terms of the proportion of time spent or dives made in different oceanographic/bathymetric domains. For both plasma and red blood cells, the models accurately predicted habitat use for animals that foraged exclusively off or on the continental shelf. The models did not perform as well in predicting habitat use for animals that foraged in both on- and off-shelf habitat; however, sample sizes for these animals were small. Concurrently collected scat, fatty acid, and dive data confirmed that the foraging differences predicted by isotopes were associated with diet differences. Stable isotope samples, dive data, and GPS location data collected from an additional 15 females during August-October 2008 validated the effective use of the models across years. Little within year variation in habitat use was indicated from the comparison between stable isotope values from plasma (representing 1-2 weeks) and red blood cells (representing the prior few months). Constructing predictive models using stable isotopes provides an effective means to assess habitat use at the population level, is inexpensive, and can be applied to other marine predators.

## Introduction

Understanding how species utilize habitat and prey resources is a key aspect of foraging ecology and necessary to assess impacts of anthropogenic and environmental influences on a population [1–4]. Characterizing foraging habitats of wide-ranging marine predators is challenging due to the difficulty of observing animals at sea. Telemetry studies have been used extensively to describe habitat use of marine predators including fish, birds, and marine mammals [5–10]. However, these studies are often limited to relatively small numbers of animals because satellite tags are expensive and typically have short sampling durations due to challenges with instrument retention[11]. In addition, due to the need for a small tag to body size ratio, significant data gaps remain in telemetry studies with respect to smaller organisms and early life history stages[12, 13].

Examining a predator’s diet can also increase our understanding of habitat use and resource requirements. Diet studies based on prey remains found in fecal samples have frequently been used to assess foraging habitat [14–16]. Fecal analysis identifies specific prey species and size of prey consumed; however, it is often associated with several known biases including differences in prey digestion rates and specific prey parts being regurgitated [17–22]. Certain prey species are underrepresented in scats because they do not have identifiable remains (e.g. invertebrates and cartilaginous fish) or have identifiable hard parts that are not ingested [23, 24]. Scats only represent feeding just prior to collection and do not provide integrated long-term foraging information[25]. Furthermore, scat analysis does not provide information on individual animals (e.g. age and sex) making intraspecific diet comparisons difficult.

Fatty acid (FA) analysis is another technique commonly used to estimate predator diet [26–28], and it is based on the knowledge that FA from prey are assimilated, in a predictable manner, into a consumer’s tissues [24, 29]. FA provide dietary information over a range of time scales depending on the tissue sampled (e.g., from several hours in blood or milk to days or weeks in fat storage sites) and level of consumption and mobilization [29, 30]. Qualitatively, differences found in FA composition of a given fat storage site among individuals indicate differences in diets [29]. Although a number of other factors must be taken into account (knowledge of predator metabolism effects, a comprehensive prey FA database), when appropriately used, FA can also produce accurate estimates of predator diets [20, 26, 29, 30].

An alternative approach to quantifying both diet and habitat use for a wide range of species is to measure stable isotope ratios of nitrogen and carbon in different tissues [31]. Because stable isotopes can be collected from large numbers of animals, don’t require animals to be recaptured and are relatively inexpensive to analyze, this approach has the potential to quantify habitat use patterns at a population level [32]. Furthermore, stable isotopes provide a method to describe habitat use for marine species that are less tractable using scat, fatty acid, or satellite data.

The stable nitrogen (^15^N/^14^N or δ^15^N) and carbon (^13^C/^12^C or δ^13^C) isotope ratios of a consumer’s tissue indicate trophic level and foraging location, respectively [33–35]. Nitrogen isotopes undergo fractionation between predator and prey, leading to an increase of ^15^N with each trophic level [36]. In marine systems, nitrogen isotope values increase predictably (~3–4‰) with each trophic level [37, 38]. Carbon isotope values are driven by the isotope values at the base of the food web, have little variation between trophic levels, and are primarily used to indicate foraging locations of animals on small and large spatial scales. General patterns in carbon isotope values are that they increase from high to middle latitudes, offshore to nearshore, and pelagic to benthic environments [38–42]. Stable isotope analysis of different tissues provides assimilated dietary information over a range of time scales due to the dissimilar isotopic turnover rates of the different tissues [43]. For example, δ^15^N and δ^13^C values of blood plasma reflect diet integrated approximately 1–2 weeks prior to collection whereas those of red blood cells (RBCs) represent the diet of the previous one or two months [44–48].

**Fig 1:**
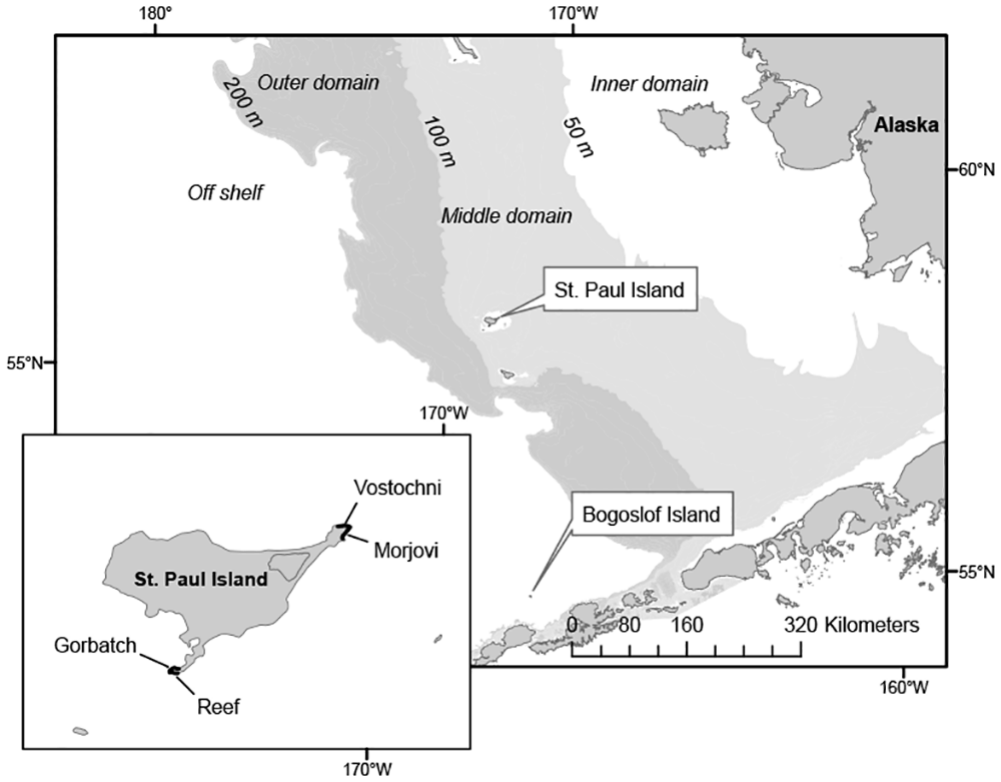
Location of northern fur seal rookeries where blood and scat samples were collected: Reef, Gorbatch, Morjovi and Vostochni rookeries on St. Paul Island and Bogoslof Island. The oceanographic domains in the Bering Sea are shown.

Northern fur seals (*Callorhinus ursinus*) from breeding colonies in Alaska are particularly well-suited subjects for evaluating predictions of foraging habitat of a top marine predator based on stable isotope analysis. Fur seal foraging habitat in the eastern Bering Sea is defined by two distinct oceanographic regions: the North Pacific continental shelf, which includes waters < 200 m (on-shelf), and the Bering Sea deep sea basin (off-shelf; Fig 1). The on-shelf region is further divided into three depth domains termed the inner (0–50 m), middle (51–100 m), and outer (101–200 m) domains (Fig 1; [49]). The marine domains are characterized by differences in water column structure, currents, and species composition [50–52].

Diet studies based on fecal analysis indicate that fur seals from different breeding colonies feed in distinct food web communities (on-shelf vs. off-shelf domains; [15, 52–54]. At-sea tracking of adult female fur seals also shows colony-specific foraging areas associated with marine domains [5, 55–57]. Dive parameters of fur seals vary depending on foraging habitat: off-shelf foragers tend to make shallow dives (typically within the first 30m) during the night-time; on-shelf foragers make deeper dives (>50m) during both day- and night-time [56, 58, 59]. These characteristics suggest that stable isotope values of northern fur seal tissues should vary in a consistent pattern within this population.

The objective of this study was to develop models that use stable isotope values of blood to predict foraging habitat of adult female northern fur seals. To assess the feasibility of these models, we compared individual δ^13^C and δ^15^N isotope values from RBCs and plasma with individual satellite location and time-depth recorder dive data collected in 2006. Independent data collected in 2008 was used to test the performance of the models and to determine whether they could account for annual differences in stable isotope values. Fecal, dive, and FA analysis were used to validate foraging habitat groupings predicted by the models. Finally, to evaluate whether observed patterns of habitat use and foraging site fidelity in individual seals were reflected in their isotopes, we compared values of plasma and RBCs, which represent diet assimilated over weeks and months, respectively.

## Materials and Methods

### Study site and sample collection

In 2006, adult female northern fur seals with pups were captured at three breeding colonies on two islands in Alaska: Bogoslof Island (n = 15; 53.9°N, 168.0°W) and Reef (n = 10; 57.1°N, 170.3°W) and Vostochni (n = 6; 57.3°N, 170.1°W) rookeries on St. Paul Island (Fig 1). In 2008, adult females were captured at Reef (n = 5) and Vostochni (n = 10) rookeries on St. Paul Island. All study sites are located close to the North Pacific continental shelf: Reef and Vostochni are located on opposite sides of St. Paul Island and Bogoslof Island is located about 200km to the southeast (Fig 1).

Adult females were captured in July and August using a hoop-net or mobile blind, transferred to a restraint board [60], and instruments were attached to the dorsal pelage using quick set epoxy. In 2006, KiwiSat 101 (Sirtrak, New Zealand) platform transmitter terminals (PTT) were used to acquire at-sea locations via the Argos satellite system. PTT transmissions were duty-cycled for 4 hours on, 4 hours off, and were programmed to start the duty cycle at 03:00 GMT and shut off after a 4-hour dry period. In 2006, dive data were collected using Mk9 (Wildlife Computers, USA) time depth recorders (TDR) programmed to sample dive depth every 5 seconds. In 2008, Mk10-AF tags (GPS and TDR combined, Wildlife Computers) recorded GPS locations continuously when at the surface and transmitted a subset of these locations via the Argos satellite system. The Mk10-AFs recorded dive depth at 1- or 5-second intervals. Very high frequency or VHF tags (Advanced Telemetry Systems, USA) were attached to all animals in both years to assist with animal recapture and instrument recovery.

Adult females were recaptured in October to recover tracking instruments and collect blood samples for isotope analysis. Blood samples were obtained from the dorsal side of the rear flipper using a 21-gauge butterfly needle and placed directly in Vacutainer tubes. Plasma and RBCs were collected from tubes with sodium heparin, an anticlotting agent that does not alter isotopic signatures [61]. The tubes were centrifuged for 10 minutes. Between 1 and 2 ml of each blood component was decanted into cryovials and stored in a -40°C freezer for later laboratory processing.

Scat samples were collected for comparison of diet among years and sites. Scats were collected independently from the study animals at Reef and Vostochni rookeries on St. Paul Island in September 2006 and on Bogoslof Island in September 2007. In September 2008, scat samples were collected at Gorbatch and Morjovi rookeries which are adjacent to Reef and Vostochni on St. Paul Island (Fig 1). Studies have indicated that fur seal diet at Gorbatch is comparable to diet at Reef and that diet at Morjovi is comparable to diet at Vostochni [15]. Scats were collected at each site on a single day. At all locations, we assumed that scats collected at breeding colonies primarily represent the diet of adult females. Scat samples were stored in plastic bags, frozen and returned to the laboratory for identification.

In 2006, milk and blubber samples were collected during recaptures for FA analysis to investigate differences in diet between on- and off-shelf foragers. FA from milk and blubber collected from a lactating fur seal that has returned from a ~7 day foraging trip should represent diet integrated over a period of days to weeks, respectively, given that females have consumed prey FA and deposited these in blubber and/or secreted milk during this period [24, 29]. Samples of milk were manually expressed after administering oxytocin intramuscularly (0.25 ml at 20 IU/ml, Butler Schein, Dublin, OH, USA) to facilitate milk letdown. A subsample was placed in chloroform containing an antioxidant and stored frozen until they could be processed and analyzed in the laboratory. A full-depth blubber sample was taken from the flank using a medical biopsy punch and stored similarly.

All work was conducted in accordance with and under the authority of the United States Marine Mammal Protection Act (National Marine Fisheries Service, NMFS Permits 782–1708). The Marine Mammal Protection Act was established in 1972 requiring all research conducted on marine mammals in the United States be done under the authority of federal permits issued by either NMFS or U.S. Fish and Wildlife Service (USFWS). All applications for a permit to conduct research on marine mammals have gone through a four-stage review process that includes: 1) agency review (either NMFS or USFWS); 2) a public notice and review period; 3) review and recommendation from the Scientific Advisers to the U.S. Marine Mammal Commission; and 4) a final action by the reviewing agency. All capture and handling activities described in this manuscript have gone through and been approved by this process. At the time when adult female NFS were captured by NMFS there was no additional requirement for review of these procedures by an institutional review board or ethics committee. In 2010, a NMFS Institutional Animal Care and Use Committee was established for the Alaska Fisheries and Northwest Fisheries science centers and the capture and handling protocols described here were reviewed and approved by this committee.

### Location and dive data analyses

Complete foraging trips covering the departure and return to a rookery were determined using both dive and location data. In 2006, animal locations were calculated by Service Argos Inc. All low-quality (class Z) locations were removed from the PTT data and location data (PTT and GPS) were filtered using an algorithm based on swimming speed, distance between successive locations, and turning angles ([62]; swim speed = 3m s^-1^). Foraging tracks were reconstructed by modeling the filtered location data using a continuous-time correlated random walk model [63]. Locations were interpolated hourly as well as at each dive in order to measure foraging effort. Only dives > 5m were included in the analysis [9, 64, 65].

Hourly and dive interpolated locations were spatially joined with bathymetric data in ArcMap (version 10.0). Three different bathymetry data sets were used to cover the extent of the data. St. Paul Island data were overlaid with NOAA shelf polygon covers (WBER_NBS and EBER_NBS) that corresponded to the five oceanographic domains and Bogoslof Island data were overlaid with data gridded to the lowest possible extent ranging from 50 m to 1500 m grids (Steve Lewis, NMFS). Oceanographic domains associated with each foraging trip were used to categorize animals by foraging strategy: on-, off-, or mixed-shelf foragers. On-shelf foragers exclusively utilized the on-shelf habitat, while off-shelf foragers traveled beyond the 200m isobaths to the off-shelf domain to forage. Mixed-shelf foragers made foraging trips to both on- and off-shelf habitat. The proportion of hours or dives made in the on-shelf habitat during each trip was then calculated as a measure of habitat use associated with each foraging strategy.

To explore diving behavior associated with each foraging strategy, we assessed mean dive depth and the proportion of nighttime dives for on-, off- and mixed-shelf foragers. Mean dive depth and standard deviation were calculated using maximum dive depths for day and night combined, averaged first by individual and then over individuals within each foraging strategy. The proportion of night time dives was determined using local sunrise and sunset times, and the R package “maptools” to categorize all dives as being either during the day or night. Mean proportion of nighttime dives was calculated for each animal and then averaged over individuals within each foraging strategy.

### Stable isotope analysis

Frozen blood samples were placed in a lyophilizer and dried for 24 to 48 hours. The dried samples were then ground into a powder and homogenized using a glass rod. Samples were weighed (1.0 ± 0.2mg) and sealed into 8 × 5 mm tin capsules and analyzed using continuous flow isotope ratio mass spectrometer (20–20 PDZ Europa) at the University of California Davis Stable Isotope Facility (Davis, CA, USA). The natural isotopic abundance in a sample is expressed in delta (δ) notation, δ^13^C or δ^15^N = 1000×[(R_sample_/R_standard_)×1], where R_sample_ and R_standard_ are the ^13^C/^12^C or ^15^N/^14^N ratios of the sample and standard, respectively. The standards are Vienna-Pee Dee Belemnite limestone (V-PDB) for carbon or atmospheric N_2_ for nitrogen. The units are expressed as parts per mil (‰).

### Habitat model

We used location, dive and blood isotope data from 2006 to construct a GLMM models to predict foraging habitat from stable isotope ratios of carbon and nitrogen from plasma and RBCs. The proportion of hours or dives made in the on- and off-shelf habitat was used to characterize the foraging habitat utilized by each animal during their foraging trips, and to compare habitat use among foraging strategies. For plasma, foraging habitat was assessed for trips that occurred within two weeks of blood collection, whereas for RBCs it was evaluated for all trips completed over the breeding season. All statistical analyses were performed using the R Program Language [66].

The number of hours or dives spent on shelf was used as a response variable and stable isotope ratios of carbon and nitrogen from plasma and RBCs were used as explanatory variables in binomial generalized linear mixed models (GLMMs). We normalized the δ^15^N values by subtracting 13 and the δ^13^C values by adding 20 to make the associated coefficients numerically more stable in the likelihood optimization. Normally distributed random intercepts for individual animals and for trip within individual animal were included in the model to account for correlation of binomial counts due to individual heterogeneity. We investigated stable isotope model structure using several models (additive, quadratic, with and without δ^13^C*δ^15^N interaction). Model selection was based on how well the chosen model fit the 2006 data and predicted the 2008 data with respect to root mean square prediction error over animals. For plasma, a binomial GLMM which included main effects for δ^13^C and δ^15^N was the best fit. The RBC model benefitted by the addition of a quadratic effect for δ^15^N. We tested a quadratic effect for δ^13^C, but it did not improve the model fit. All GLMMs were fitted with lme4 package in the R statistical environment [67]. We also tested for nonparametric curvature with a generalized additive mixed model (GAMM) containing the previous mentioned random effects; the GAMM over-fit the data and produced poor predictions during trial runs with additional data. Finally, data from 2008 were used to test the model’s performance in predicting habitat use by the animals, based solely on their blood stable isotope data.

### Scat analysis

The scat samples collected from rookeries in 2006 and 2008 were used to determine if the habitat differences predicted by the stable isotope data were associated with differences in diet among the breeding colonies. In the laboratory, scat samples were thawed and rinsed in nested sieves (2.0, 1.0, and 0.5 mm) to recover prey remains. Fish remains (bones and otoliths) and cephalopod parts (eye lenses, statoliths and beaks) were identified to the lowest possible taxon using reference collection specimens. The relative importance of each prey species was estimated using frequency of occurrence (FO). We calculated FO by dividing the number of scats in which a prey taxon occurred by the total number of scats with identifiable prey remains.

### Fatty acid analysis

Lipids from milk and blubber samples collected in 2006 were quantitatively extracted from blubber and milk, and FA methyl esters were prepared, identified, and analyzed according to Iverson et al. [68]. We used temperature-programmed gas liquid chromatography on a Varian Capillary FID gas chromatograph fitted with a 30 m x 0.25 mm id. column coated with 50% cyanopropyl polysiloxane (0.25μ film thickness; Agilent Technologies DB-23; Palo Alto, CA) and linked to a computerized integration system (Varian Galaxie software). FA are expressed using the shorthand nomenclature of A:Bn-X, where A represents the number of carbon atoms, B the number of double bonds and X the position of the double bond closest to the terminal methyl group.

Seventeen FA from blubber and milk which best represent major dietary FA and are most variable were transformed using the centered logratio transformation: *x*_t_ = log(*x*_i_/*g*(*x*)), where *x*_i_ is a given FA expressed a percent of total, *g*(*x*) is the geometric mean of the FA data within each individual and *x*_t_ represents the transformed FA data [69]. Hierarchical cluster analysis of the 17 FA was used to determine whether there was a relationship between FA signatures and foraging habitat. The cluster analysis was conducted using squared Euclidian distance [70] as a measure of similarity among individuals and Ward’s method to compare cluster distances [71].

## Results

### Location and dive data analysis

In 2006, Argos location data were collected from 31 females and dive data were collected from 28 females (Table 1). In 2008, location data were collected from 15 females and dive data were collected from 14 females (Table 1). The satellite tags on one animal in 2006 and the GPS tags on 3 animals in 2008 stopped working several weeks before recapture but were still included in the analyses because most of the satellite tracking study period was represented.

**Table 1:** Number of northern fur seal stable isotope samples, location data and dive data collected in 2006 and 2008.

Location	Plasma	RBC	location	dive
**2006**				
St. Paul (Reef)	10	10	10	8
St. Paul (Vostochni)	6	6	6	5
Bogoslof	15	15	15	15
**2008**				
St. Paul (Reef)	5	5	5	4
St. Paul (Vostochni)	10	10	10	10

**Fig 2:**
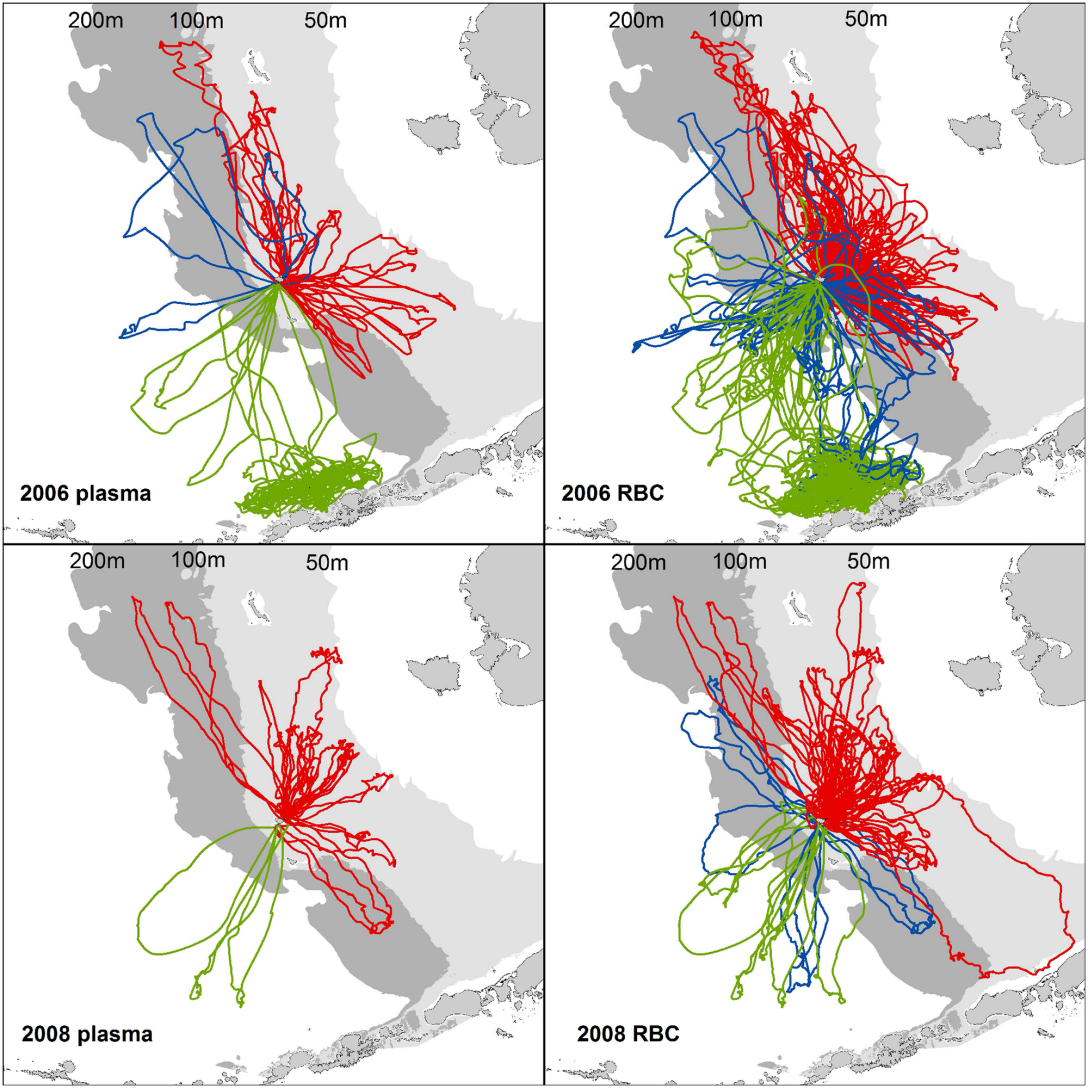
Trackline data for adult female northern fur seals tagged in 2006 and 2008. Red, green and blue lines indicate animals categorized as on-, off-, or mixed-shelf foragers, respectively. Plasma includes any trips which occurred within two weeks of blood collection and red blood cells represent all trips preceding the blood sample collection.

During both years, animals from each breeding site made foraging trips to distinct habitats in the Bering Sea that allowed categorization of their foraging strategies (Fig 2, Table 2). In 2006, Bogoslof Island females foraged almost exclusively off-shelf, dove at night (night-time dives = 98.4%, SD = 3.7%), and made shallow dives (mean dive depth = 13.6m, SD = 1.6m). Bogoslof Island trips were very short, 23% had one or less satellite locations per trip and were excluded from developing the habitat model. St. Paul Island females from Vostochni foraged exclusively on-shelf, predominately in the middle-shelf domain (Fig 1). Vostochni females mostly foraged at night (night-time dives = 87.9%, SD = 7.2%) and had more variable dive depths (mean = 28.4m, SD = 2.5m). Of the 8 Reef animals with dive data, 4 foraged exclusively on-shelf, 2 were off-shelf and 2 were mixed-shelf foragers. Reef off-shelf foragers primarily foraged at night (94.7%, SD = 6.6%) at relatively shallow depths (mean dive depth = 15.8m, SD = 6.2m). Reef on-shelf foragers had fewer night time dives (80.5%, SD = 4.4%) and a mean dive depth of 29.6m (SD = 3.8m). The mixed-shelf foragers all switched from off-shelf to on-shelf foraging at different times during the study period. Reef mixed-shelf foragers had 86.7% (SD = 4.5%) night-time dives and a mean dive depth of 20.4m (SD = 4.4m).

**Table 2:** Percentage of hours and dives made on the continental shelf by northern fur seals characterized as having on-, mixed-, and off-shelf foraging strategies.

Location (Foraging strategy)	Animals (n)	% On-shelf hours Mean (SD)	% On-shelf dives Mean (SD)
**2006**			
**St. Paul (Vostochni)**			
On-shelf	6(location) 5(dive)	100	100
**St. Paul (Reef)**			
On-shelf	4	100	100
Mixed	3(location) 2(dive)	53.6 (21.7)	28.2 (0.4)
Off-shelf	3(location) 2(dive)	26.4 (9.4)	8.4 (2.1)
**Bogoslof**			
Off-shelf	15	13.0 (7.7)	9.9 (8.8)
**2008**			
**St. Paul (Vostochni)**			
On-shelf	10	100	100
**St. Paul (Reef)**			
Mixed	2	76.5 (15.9)	69.3 (24.6)
Off-shelf	3(location) 2(dive)	31.5 (3.8)	28.5 (31.5)

Sample sizes include number of animals with location and dive data.

St. Paul Island foraging patterns in 2008 were similar to 2006 (Table 2). All Vostochni females foraged on shelf, primarily in the middle-shelf domain at night (night-time dives = 71.9%, SD = 6.6%) with a mean dive depth of 23.4m (SD = 5.6m). Two Reef females were off-shelf foragers and two were mixed-shelf foragers. One of the off-shelf animals foraged almost exclusively at night and the other foraged during both day and night (mean night time dives = 74.2%, SD = 23.8%), but both foraged at relatively shallow depths (mean dive depth = 11.2m, SD = 1.2m). As in 2006, the mixed-shelf foragers all switched from foraging off-shelf to foraging on-shelf. A mean of 78.1% (SD = 2.4%) of the dives of the mixed-shelf foragers occurred at night with a mean dive depth of 27.0m (SD = 14.9m).

### Stable isotope analysis

**Fig 3:**
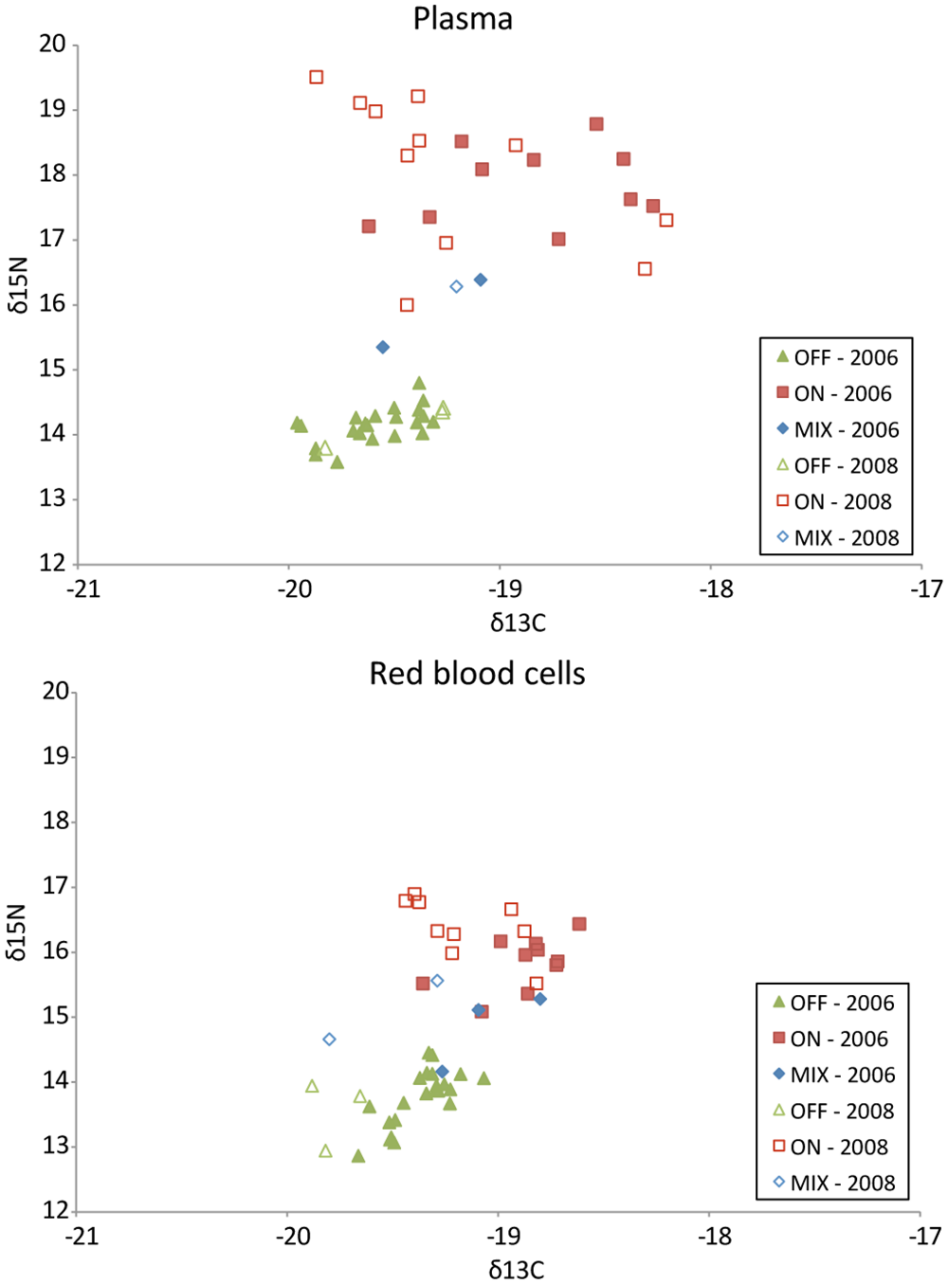
Individual δ^15^N and δ^13^C values for plasma and red blood cell tisues collected from adult female northern fur seals at Bogoslof and St. Paul Island rookeries during 2006 and 2008. On-shelf foragers (ON) exclusively utilized < 200m depth, off-shelf foragers (OFF) traveled beyond the 200m isobaths to forage and mixed-shelf foragers (MIX) made foraging trips to both on- and off-shelf habitat.

Plasma and RBC samples were collected for stable isotope analysis from 31 females in 2006 and 15 females in 2008 that made foraging trips to distinct habitats in the Bering Sea (Table 1, Fig 2). In 2006, isotopic signatures of plasma ranged from -19.96 to -18.27 for δ^13^C and 13.58 to 18.79 for δ^15^N; for RBCs, values ranged from -18.62 to -19.66 for δ^13^C and 12.86 to 16.82 for δ^15^N. In 2008, isotopic signatures of plasma ranged from -19.87 to -18.21 for δ^13^C and 13.80 to 19.51 for δ^15^N; for RBCs, values ranged from -19.88 to -18.82 for δ^13^C and 12.94 to 16.90 for δ^15^N. The groupings suggested by plotting the stable isotope values with foraging habitats suggest relationships between these variables for both plasma and RBCs (Fig 3).

**Fig 4:**
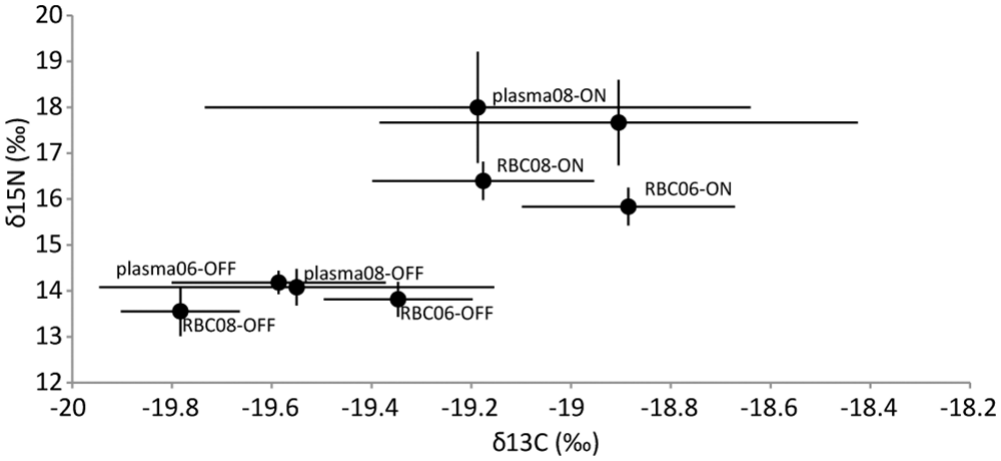
Mean (±SD) δ^15^N and δ^13^C values for plasma and red blood cell (RBC) tissues collected from adult female northern fur seals in 2006 and 2008. On-shelf foragers (ON) exclusively utilized < 200m depth and off-shelf foragers (OFF) traveled beyond the 200m isobaths to forage.

For plasma and RBCs, δ^13^C and δ^15^N isotope values of animals foraging on-shelf were higher in comparison to off-shelf foragers (Fig 4; p < 0.001 from t-tests). For plasma, there were no differences in δ^13^C and δ^15^N stable isotope values between years when data was divided by foraging strategy (p = 0.074 from t-tests). However, for RBC the mean δ^13^C values were lower in 2008 than 2006 for on- and off-shelf foragers (p = 0.002, t-tests) and δ^15^N values were higher in 2008 than 2006 for on-shelf foragers (p = 0.006, t-tests). The only difference in δ^13^C isotope values between sample types was that in 2006 off-shore foragers had lower δ^13^C values for plasma than RBCs (p = 0.003, t-test). In contrast, δ^15^N values of plasma were higher than those from RBCs for both years and all foraging strategies (p = 0.021, t-tests), with the exception of 2008 off-shore foragers (p = 0.110, t-test).

### Habitat model

**Fig 5:**
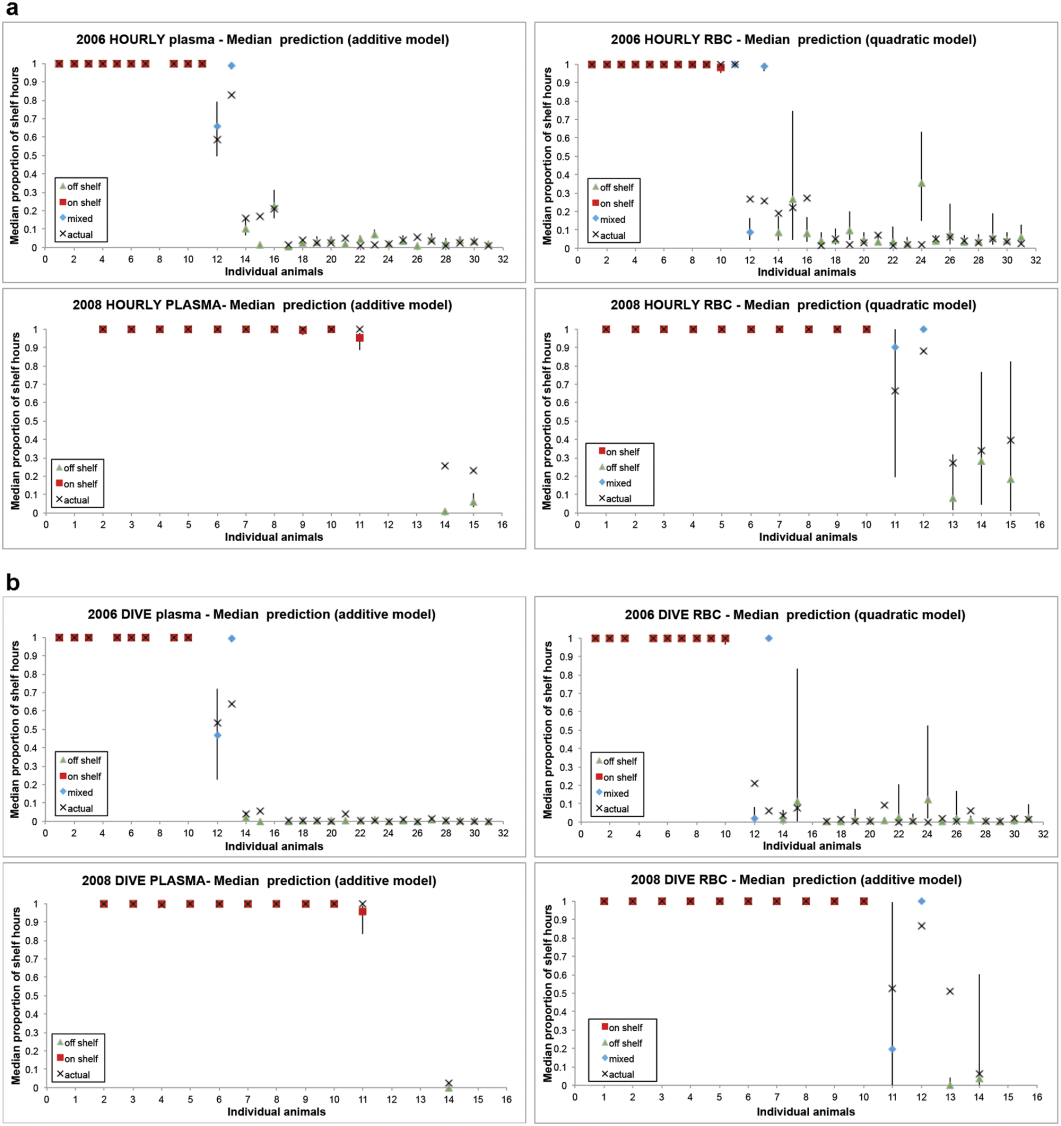
Predicted and actual proportion of hours (a) and dives (b) individual northern fur seals spent on the shelf with 95% CI (from generalized linear mixed model) for red blood cells & plasma). Red, green and blue lines indicate animals categorized as on-, off-, or in mixed-shelf foragers, respectively.

The GLMM model derived from the stable isotope data collected from plasma in 2006 accurately predicted habitat use (the proportion of hours and dives that occurred on-shelf) for animals categorized as having either on- or off-shelf foraging strategies (Fig 5a and 5b). The model predicted habitat use for mixed-shelf foragers with less accuracy, however there were only two animals with plasma samples which utilized this strategy in 2006. Habitat use for one of the mixed-shelf foragers was predicted accurately and the other spent more hours and made more dives off-shelf than predicted. For RBC, the model correctly predicted habitat use for all on-shelf foragers and all but one off-shelf foragers (Fig 5a and 5b). As with plasma, the RBC model predicted habitat use for mixed-shelf foragers with less accuracy than on- and off-shelf foragers (Fig 5a and 5b).

When the model was tested using the stable isotope data from plasma collected in 2008, it correctly distinguished habitat use for almost all on- and off-shelf foragers, despite differences in δ^15^N and δ^13^C isotope values between years, suggesting the model is robust to yearly environmental shifts in stable isotope values (Fig 5a and 5b). Although classified correctly, off-shelf foragers were predicted to spend fewer hours off-shelf than observed. Unfortunately, no plasma samples were collected from the mixed-shelf foragers in 2008 which limited model testing for mixed-shelf foragers in that year. Using the stable isotope data collected from RBCs in 2008, the model correctly predicted habitat use for all on-shelf foragers (Fig 5a and 5b). For the off-shelf foragers, the model correctly predicted proportion of hours and dives on-shelf from RBCs with the exception of one animal that dove on-shelf less frequently than predicted. There were only 2 mixed shelf foragers in 2008; the RBC model correctly predicted proportion of hours and dives for one of the two.

### Scat analysis

We collected 206 scats with identifiable prey remains (Table 3). Our results were consistent with previous diet studies[15, 54]. Walleye pollock (*Gadus chalcogrammus*) was the dominant prey item on St. Paul Island rookeries in both years (Table 3). Scats from Vostochni (2006) and Morjovi (2008) contained almost exclusively on-shelf prey species, such as walleye pollock, Pacific herring (*Clupea harengus*), Pacific sand lance (*Ammodytes hexapterus*), and sand fish (*Trichodon trichodon*). There was little difference in species assemblage and FO between years with the exception of Pacific herring which had a much higher FO in 2006 (27.03%) than 2008 (7.32%).

**Table 3:** Percent frequency of occurrence (%FO > 5.0%) of prey taxa retrieved from northern fur seal fecal samples collected at rookeries from 2006–2008.

Prey taxa	Reef	Vostochni	Bogoslof	Gorbatch	Morjovi
Year	2006	2006	2007	2008	2008
	(*n* = 28)	(*n* = 74)	(*n* = 41)	(*n* = 22)	(*n* = 41)
*Leuroglossus schmidti* (Northern smoothtongue)			**73.2**	**18.2**	
*Gadus chalcogrammus* (Walleye Pollock)	**89.3**	**68.9**	9.8	**31.8**	**58.5**
Gonatid squid	3.6	5.4	**73.2**	**18.2**	
*Clupea harengus* (Pacific herring)	**14.3**	**27.0**		4.6	7.3
*Oncorhynchus* spp. (Pacific salmon)	**17.9**	**18.9**		**22.7**	**12.2**
Gadid	3.6	**25.7**	2.4	**40.9**	**22.0**
Myctophid spp. (Lanternfish)	**10.7**		**17.1**	4.6	
*Pleurogrammus monopterygius* (Atka mackerel)	**14.3**	6.8	2.4	**18.2**	4.9
*Ammodytes hexapterus* (Pacific sand lance)		5.4		**13.6**	
*Gasterosteus aculeatus* (Three-spined stickleback)		5.4			
*Hemilepidotus* spp. (Irish lord spp.)		5.4			
*Gadus macrocephalus* (Pacific cod)					9.8
*Trichodon trichodon* (Pacific sandfish)					7.3

*n* indicates the number of samples that had identifiable prey remains. Bold numbers indicate prey taxa with %FO > 10.0%.

Reef (2006) and Gorbatch (2008) scats had occurrences of both on-shelf species (walleye pollock, Pacific herring, Pacific sand lance) and off-shelf species (mycytopids and gonatid squids incuding *Gonatopsis borealis*, *Berryteuthis magister*, *G*. *onyx*, and *G*. *tinro*) prey species. Reef and Gorbatch scats also had relatively high occurrences of Atka mackerel (*Pleurogrammus monopterygius*) which occurs along the edge of the continental shelf edge (Table 2). Walleye pollock FO was lower in scats collected on Gorbatch (31.82%) than Reef (89.29), although Gorbatch scats had a higher FO of unidentified Gadids (40.91%) than Reef (3.57%). We assume the unidentified gadids in scats collected from Gorbatch were walleye pollock because no other gadid species were identified in those scats. Scats collected from animals on Bogoslof Island (2007) had high occurrences of off-shelf species, including gonatid squid (73.2%), northern smoothtongue (*Leuroglossus schmidti*; 73.2%) and mycytophids (17.1%).

### Fatty acid analysis

**Fig 6:**
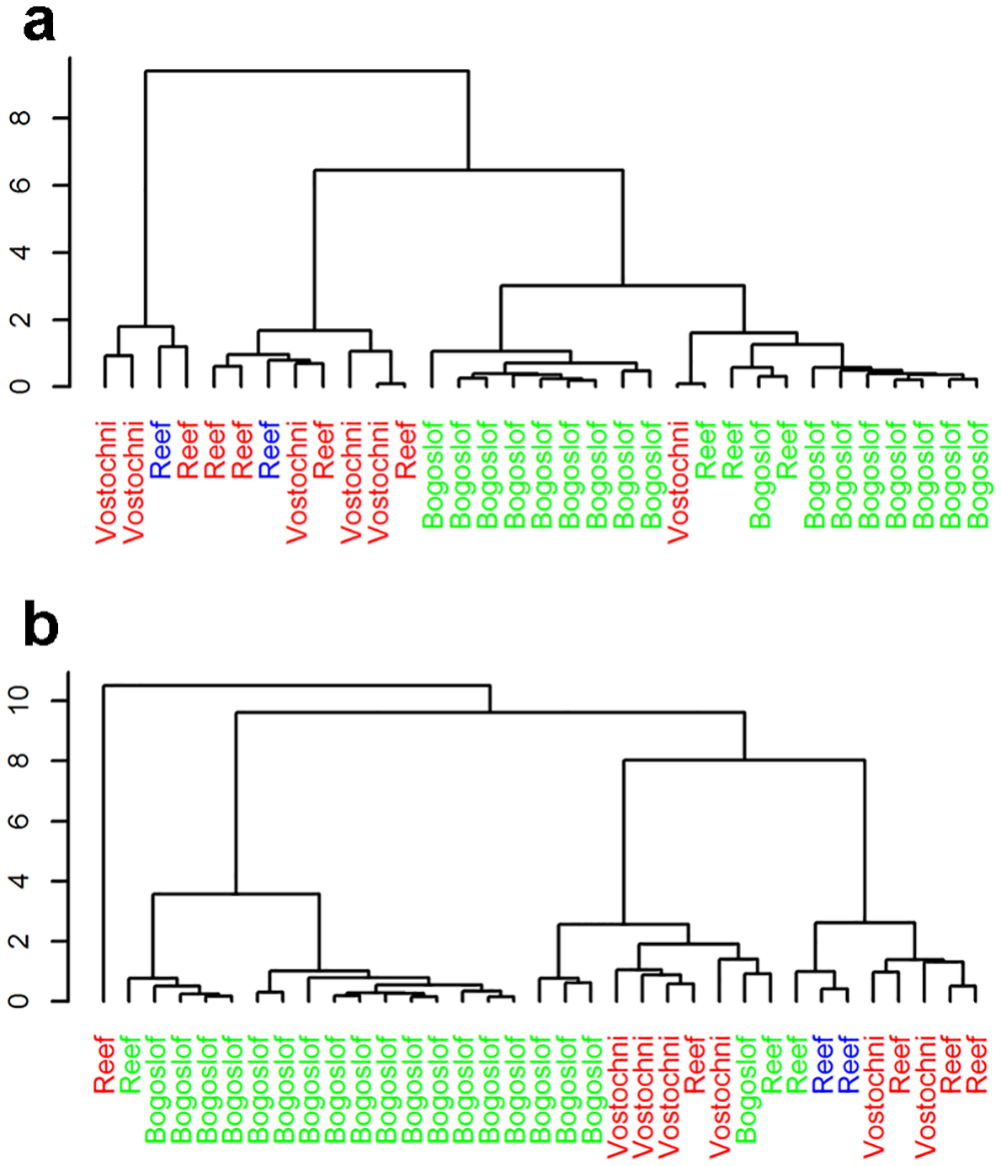
Dendograms from a hierarchical cluster analysis of 17 fatty acids from northern fur seal blubber (a) and milk (b) samples. Red, green, and blue labels indicate animals categorized as on-, off-, or mixed-shelf foragers, respectively.

Cluster analysis of individual blubber FA separated individuals into three distinct groupings: two groups were categorized as on-shelf foragers and one group was categorized as off-shelf foragers (Fig 6a). The two on-shelf groupings included animals from both Reef and Vostochni. Only one on-shelf animal from Vostochni clustered in the off-shelf grouping and mixed-shelf foragers clustered with on-shelf foragers. Cluster analysis for milk also generally separated the St. Paul Island from Bogoslof Island animals (Fig 6b). However, the cluster analysis of milk FA did not separate on-, mixed-, and off-shelf foragers as clearly as blubber FA.

## Discussion

Stable carbon and nitrogen isotope values have been used to describe foraging behavior and habitat use for a wide range of organisms [32, 38, 72]. However, only a few studies have used stable isotopes to develop predictive habitat use models. For example, δ^13^C and δ^15^N values from skin tissue collected from big brown bats (*Eptesicus fuscus*) were used to predict ecogeographic regions important to the species [73]. Using δ^13^C and δ^15^N values taken from plasma and RBCs, we developed innovative models to predict on- and off-shelf foraging habitat for adult female northern fur seals. Moreover, testing of the models with an additional year of sampling data validated that stable isotope values from RBCs and plasma can be used to accurately predict individual habitat use of a top marine predator. This approach provides the opportunity to quantify habitat use for large sample sizes of top marine predators at relatively low cost and assess habitat use for species in which other methods cannot be applied.

### Location and dive data analysis

Northern fur seals in this study foraged in distinct habitats of the Bering Sea and, based on the oceanographic domains encountered during their foraging trips, were categorized as on-, mixed-, and off-shelf foragers. These results are corroborated by previous studies, which showed that females from Vostochni and Morjovi forage on-shelf, Reef and Gorbatch animals utilize both on- and off-shelf habitat, and adult females from Bogoslof Island forage almost exclusively in off-shelf waters [5, 55, 59, 74, 75]. Differences among categories of foraging strategy were reflected in two measures of habitat use: proportion of hours and dives in the on-shelf habitat. Furthermore, the dive depths and timing of diving within the diurnal cycle that we observed were consistent with previously reported on- and off-shelf northern fur seal dive behavior studies [9, 56, 59]. In both years, females foraging off-shelf from Reef and Bogoslof rookeries predominately dove during the night-time at shallow depths (<30m). On-shelf foragers from Reef and Vostochni rookeries dove during both day and night time to deeper depths than off-shelf foragers. Differences in dive behavior among northern fur seals with different foraging strategies corresponds to differences in the behavior of prey species consumed in these habitats. Most off-shelf prey species (northern smoothtongue, myctophids, and Gonatid squid) are located indeep waters (200–1000m) and migrate to the surface at night to feed [76, 77]. In comparison, many on-shelf prey (e.g. Pacific sand lance) rarely occur at depths > 200m [78].

### Stable isotope analysis

For both 2006 and 2008, mean δ^15^N and δ^13^C values of plasma and RBCs from animals foraging on-shelf were higher than off-shelf foragers. Previous pinniped studies found higher δ^15^N and δ^13^C values to be associated with on-shelf foraging. For example, Waite [79] found Steller sea lions *(Eumetopias jubatus)* foraging in nearshore benthic environment had significantly higher δ^15^N and δ^13^C values compared to northern fur seals that were foraging in predominantly off-shore pelagic environments. Comparison of bone collagen δ^13^C values among four species of sympatric pinnipeds in the northeast Pacific also found that nearshore foragers had higher δ^13^C values compared to species that forage pelagically off-shore along the continental shelf edge [80, 81].

The δ^13^C values were similar for plasma and RBCs in both 2006 and 2008 when tissues were grouped by foraging strategy. The only significant difference was that the δ^13^C values from plasma were lower than those from RBCs for 2006 off-shore foragers. Except for 2008 off-shore foragers, the δ^15^N values from plasma were all significantly higher compared with those from the RBCs. Zeppelin and Orr found no difference in mean δ^13^C values between plasma and RBCs from northern fur seals and that mean δ^15^N values of plasma were higher than those from RBCs; however, these animals were not divided by foraging strategy [82]. For captive northern fur seals, Kurle also found that the δ^15^N values from plasma were higher than those from RBCs and δ^13^C values from plasma were lower than those from RBCs[83].

### Habitat model

Our models accurately predicted on- and off-shelf foraging habitat of female fur seals using δ^13^C and δ^15^N isotope values from both plasma and RBCs. Most of the females sampled in our study utilized the same foraging habitat (on- or off-shelf) for the duration of the study period. Previous studies that tracked female fur seals for multiple trips also found high fidelity to foraging habitat [55, 56]. However, 3 of the 31 females in 2006 and 2 of the 15 females in 2008 were classified as mixed-shelf foragers, having utilized on- and off-shelf habitat on different foraging trips. Intermediate stable isotope values have previously been reported for marine mammals that forage on prey at both high and low trophic levels [84, 85]. Similarly, we found that mixed-shelf foragers had δ^13^C and δ^15^N values intermediate to the two other foraging strategies. However, our model predicted foraging habitat for mixed-shelf foragers with less precision, likely due to small sample size.

The different temporal scales associated with the stable isotope values from RBCs and plasma provide an opportunity to assess the model’s ability to detect changes in an animal’s habitat use. For the most part, this was difficult to accomplish because females generally utilized the same foraging habitat for the duration of the study. However, one of the mixed-shelf foragers (individual 11, 2008) switched from off- to on-shelf habitat at a time period that corresponded to isotopic turnover of RBC and plasma tissues (1 to 2 months vs. 1 to 2 weeks prior to tissue collection, respectively). To detect a switch from off- to on- one would expect to see lower δ^13^C values from RBC than plasma, as on-shelf δ^13^C values are generally higher than those from off-shelf habitat. The shift in habitat could also impact δ^15^N values if it was associated with a change in diet. That is what we observed with the stable isotope data from individual 11, based upon these isotope data, the model correctly predicted the habitat use switch for this animal and the model predictions matched what we observed from the tracking data. (Fig 5a and 5b). However, additional data are necessary to adequately assess how accurately the model could distinguish a change in foraging between the time frames associated with plasma and RBCs.

Most of the off-shelf foragers used to build the models were from Bogoslof Island (n = 15); only 3 were from Reef rookery on St. Paul Island. When we tested the model with the 2008 data, our model consistently predicted that the off-shelf foragers utilized less on-shelf habitat than was actually observed. This finding is likely due to the fact that these animals were all from St. Paul and must transit across the shelf to reach the off-shelf habitat (in comparison with Bogoslof animals which have immediate access to off-shelf habitat). As a result, the calculation of observed habitat use by these St. Paul animals incorporates the transit period, while the modeled results from blood SI signatures suggest that these animals are feeding off-shelf and not during the transit to the island through on-shelf habitat. In addition, the predicted proportion of on-shelf dives was lower than the observed proportion of on-shelf hours for these animals, further suggesting foraging is predominately occurring off-shelf. This conclusion is supported by telemetry data; Benoit-Bird et al [86] found that females from St. Paul Island that went to off-shelf habitat did not exhibit foraging behavior on the return portion of their trips.

### Diet based on scat and fatty acid analyses

Higher δ^15^N values from on-shelf foragers indicate they are consuming higher trophic level prey than off-shelf foragers [37, 38]. In this study, mean δ^15^N values from plasma for on-shelf foragers were 1.7‰ higher in 2006 and 3.9‰ higher in 2008 compared to off-shelf foragers. Mean δ^15^N values from RBCs for on-shelf foragers were 2.0‰ higher in 2006 and 2.8‰ higher in 2008 than off-shelf foragers. Fecal samples collected from rookeries where fur seals foraged on-shelf (Vostochni and Morjovi) had highest occurrence of walleye pollock, but also contained Pacific herring, Pacific sand lance and sand fish. Fecal samples from Bogoslof Island, where fur seals foraged exclusively off-shelf had highest occurrences of myctophids, northern smoothtongue and gonatid squids. Consistent with these results, δ^15^N values of larger fish found in on-shelf habitat (e.g. adult pollock and Pacific herring) are higher than prey species commonly found in the off-shelf habitat (e.g. northern smoothtongue and squid; [25, 83, 87–89]).

In this study, stable isotopes did not provide information on specific prey consumed by northern fur seals, but served as an index to examine variation in diet and foraging location. Recent development of isotopic mixed models has made it possible to estimate the proportional contributions of specific prey in a predator’s diet, even when the number of prey species is large [90]. However, these models require a reference library of prey isotope values collected from the study region as well as an understanding of the isotopic fractionations that occur between prey and predator tissues. Even without knowing the specific prey consumed, our models provide valuable ecological information on both the individual and population level.

As with stable isotopes, fatty acid analysis requires a reference library of prey collected from the study region in order to determine specific prey species consumed. Because no prey were collected for this study, we used the fatty acid data qualitatively to determine whether differences in stable isotopes were associated with diet differences. Milk FA patterns are known to differ between northern fur seals foraging on-shelf and those foraging off-shelf due to differences in diet [59]. Cluster analysis of blubber FA separated on-shelf from off-shelf foragers indicating that the stable isotope patterns observed in our study were related to diet differences. Cluster analysis of milk FA separated fur seals by island but did not clearly divide animals by foraging strategy. Several confounding factors could contribute to differences in classifications using blubber vs milk fatty acids. In pinnipeds, blubber fatty acids generally represent an integration of dietary intake over weeks to months. However in a lactating otariid this process is complicated by the intermittent foraging patterns of females. Fatty acids consumed during foraging trips would be deposited in both the mammary gland (likely first priority; Iverson 1993) and the blubber, and the degree to which each tissue accumulates dietary fatty acids will depend on the length of the trips and subsequent suckling (emptying of the mammary gland). Foraging trip durations of females in this study were shown to be remarkably different between islands, with trips lasting 7–9 days from St. Paul and 1–2 days from Bogoslof, while suckling bouts were of similar durations[91]. The behavioral differences between islands likely influenced fatty acid accumulation in the two tissues, confounding the ability of milk FA to resolve animal foraging strategies.

The Pribilof Islands (St. Paul and St. George Islands, Alaska), have the highest abundance of breeding fur seals throughout the range and have undergone a substantial decline in the past decade [92]. Conversely, on Bogoslof Island, the population has increased exponentially since fur seals colonized the island in 1980 [75]. In 1988 northern fur seals were listed as ‘depleted’ under the Marine Mammal Protection Act. Monitoring population level changes in foraging behavior and habitat use over time is critical for determining demographic responses to anthropogenic and environmental effects, especially for a declining species such as the northern fur seal. The use of stable isotope analysis presented in this study represents a powerful approach to increase our understanding of foraging behavior and examine variation over time on a population level for northern fur seals as well as other marine predators.

There is a continued need for more controlled isotope studies to better understand many of the biological, ecological and biogeochemical factors that influence isotope ratios. Stable isotopes can be collected from large numbers of animals, don’t require animals to be recaptured and are relatively inexpensive to analyze; by continuing to compare individual isotopic information with high-resolution tracking information our model can be refined to detect annual oceanographic changes. Environmental and physical features of the shelf domains vary from year to year and will likely affect species distributions. Future work should also include prey collection and isotopic analysis for the development of isotopic mixed models to estimate proportions of specific prey consumed.

## Supporting Information

S1 Dataset Isotope data used to construct the GLMM model. (XLS)

S2 Dataset Proportion of hours and dives individual northern fur seals spent on the continental shelf in 2008. (XLS)

## References

[1] Boyd IL, Murray AWA. Monitoring a marine ecosystem using responses of upper trophic level predators. J Anim Ecol. 2001;70:747–60.

[2] Reid K, Croxall JP. Environmental response of upper trophic-level predators reveals a system change in an Antarctic marine ecosystem. Proc Biol Sci. 2001;268(1465):377–84. 11270434 10.1098/rspb.2000.1371 PMC1088617

[3] Arim M, Naya DE. Pinniped diets inferred from scats: analysis of biases in prey occurrence. Can J Zool. 2003;81:67–73.

[4] Wilson RP, Grémillet D, Syder J, Kierspel MAM, Garthe S, Weimerskirch H, et al Remote-sensing systems and seabirds: their use, abuse and potential for measuring marine environmental variables. Mar Ecol Prog Ser. 2002;228:241–61.

[5] Robson BW, Goebel ME, Baker JD, Ream RR. Separation of foraging habitat among breeding sites of a colonial marine predator, the northern fur seal (*Callorhinus ursinus*). Can J Zool. 2004;82:20–9.

[6] Ream RR, Sterling JT, Loughlin TR. Oceanographic features related to northern fur seal migratory movements. Deep-Sea Res Part 2 Top Stud Oceanogr. 2005;52:823–43.

[7] Aarts G, MacKenzie M, McConnell B, Fedak M, Matthiopoulos J. Estimating space-use and habitat preference from wildlife telemetry data. Ecography. 2008;31:140–60.

[8] Gilmour ME, Schreiber EA, Dearborn DC. Satellite telemetry of Great Frigatebirds *Fregata minor* rearing chicks on Tern Island, North Central Pacific Ocean. Mar Ornithol. 2012;40:17–23.

[9] Kuhn CE, Tremblay Y, Ream RR, Gelatt TS. Coupling GPS tracking with dive behavior to examine the relationship between foraging strategy and fine-scale movements of northern fur seals. Endanger Spec Res. 2010;12:125–39.

[10] Cunjak RA, Roussel JM, Gray MA, Dietrich JP, Cartwright DF, Munkittrick KR, et al Using stable isotope analysis with telemetry or mark-recapture data to identify fish movement and foraging. Oecologia. 2005;144:636–46. 15959824 10.1007/s00442-005-0101-9

[11] Rutz C, Hays GC. New frontiers in biologging science. Biol Lett. 2009;5:289–92. 10.1098/rsbl.2009.0089 19324624 PMC2679933

[12] Hazen EL, Maxwell SM, Bailey H, Bograd SJ, Hamann M, Gaspar P, et al Ontogeny in marine tagging and tracking science: technologies and data gaps. Mar Ecol Prog Ser. 2012;457:221–40.

[13] MA F, Lovell P, McConnell BJ, Hunter C. Overcoming the constraints of long range radio telemetry from animals: getting more useful data from smaller packages. Integr Comp Biol. 2002(42):1–10. 21708689 10.1093/icb/42.1.3

[14] Sinclair EH, Loughlin TR, Pearcy W. Prey selection by northern fur seals (*Callorhinus ursinus*) in the eastern Bering Sea. Fish Bull (Wash DC). 1994;92:132–56.

[15] Zeppelin TK, Ream RR. Foraging habitats based on the diet of female northern fur seals (*Callorhinus ursinus*) on the Pribilof Islands, Alaska. J Zool (1987). 2006;270:565–76.

[16] Iversen M, Aars J, Haug T, Alsos IG, Lydersen C, Bachmann L, et al The diet of polar bears (*Ursus maritimus*) from Svalbard, Norway, inferred from scat analysis. Polar Biol. 2013;36:561–71.

[17] Bigg MA, Fawcett I. Two biases in diet determination of northern fur seals (*Callorhinus ursinus*) In: Beddington J, Beverton R, Lavigne D, editors. Marine mammals and fisheries. London: George Allen and Unwin Ltd.; 1985 p. 284–91.

[18] Pierce GJ, Boyle PR. A review of methods for diet analysis in piscivorous marine mammals In: Barnes M, editor. Oceanography and marine biology: an annual review. Vol 29 Aberdeen: Aberdeen University Press; 1991 p. 409–86.

[19] Bowen WD. Reconstruction of pinniped diets: accounting for complete digestion of otoliths and cephalopod beaks. Can J Fish Aquat Sci. 2000;57:898–905.

[20] Bowen WD, Iverson SJ. Methods of estimating marine mammal diets: a review of validation experiments and sources of bias and uncertainty. Mar Mamm Sci. 2013;29(4):719–54.

[21] Gudmundson CJ, Zeppelin TK, Ream RR. Comparison of two methodologies for determining diet in northern fur seals (*Callorhinus ursinus*). Fish Bull (Wash DC). 2006;104(3):445–55.

[22] Tollit DJ, Wong M, Winship AJ, Rosen DAS, Trites A. Quantifying errors associated with using prey skeletal structures from fecal samples to determine the diet of Steller's sea lion (*Eumetopias jubatus*). Mar Mamm Sci. 2003;19(4):724–44.

[23] Tollit DJ, Schulze AD, Trites AW, Olesiuk P, Crockford SJ, Gelatt TS, et al Development and application of DNA techniques for validating and improving pinniped diet estimates. Ecological Applications 2009;19(4):889–905. 19544732 10.1890/07-1701.1

[24] Tollit DJ, Pierce GJ, Hobson KA, Bowen WD, Iverson SJ. Diet In: Boyd IL, Bowen WD, Iverson SJ, editors. Marine mammal ecology and conservation: a handbook of techniques. Oxford: Oxford University Press; 2010 p. 165–90.

[25] Hobson KA, Sease JL, Merrick RL, Piatt JF. Investigating trophic relationships of pinnipeds in Alaska and Washington using stable isotope ratios of nitrogen and carbon. Mar Mamm Sci. 1997;13(1):114–32.

[26] Iverson SJ, Field C, Bowen WD, Blanchard W. Quantitative fatty acid signature analysis: a new method of estimating predator diets. Ecol Monogr. 2004;74:211–35.

[27] Iverson SJ, Springer AM, Kitaysky AS. Seabirds as indicators of food web structure and ecosystem variability: qualitative and quantitative diet analyses using fatty acids. Mar Ecol Prog Ser. 2007;352:235–44.

[28] Thiemann GW, Iverson SJ, Stirling I. Polar bear diets and arctic marine food webs: insights from fatty acid analysis. Ecol Monogr. 2008;78(4):591–613.

[29] Iverson SJ. Tracing aquatic food webs using fatty acids: from qualitative indicators to quantitative determination In: Kainz M, Brett MT, Arts MT, editors. Lipids in aquatic ecosystems. New York: Springer; 2009 p. 281–308.

[30] Tollit DJ, Pierce GJ, Hobson KA, Bowen WD, Iverson SJ. Measurement of diet in marine mammals In: Boyd IL, Bowen WD, Iverson SJ, editors. Marine mammal ecology and conservation: a handbook of techniques. Oxford: Oxford University Press; 2010 p. 191–221.

[31] Crawford K, McDonald RA, Bearhop S. Applications of stable isotope techniques to the ecology of mammals. Mamm Rev. 2008;38:87–107.

[32] Rubenstein DR, Hobson KA. From birds to butterflies: animal movement patterns and stable isotopes. Trends Ecol Evol. 2004;19(5):256–63. 16701265 10.1016/j.tree.2004.03.017

[33] DeNiro MJ, Epstein S. Influence of diet on the distribution of nitrogen isotopes in animals. Geochim Cosmochim Acta. 1981;45:341–51.

[34] Hobson KA. Isotopic tracking of migrant wildlife In: Michener RH, Lajtha K, editors. Stable isotopes in ecology and environmental science. 2nd ed Malden (MA): Blackwell Publishing; 2007 p. 155–75.

[35] Michener RH, Kaufman L. Stable isotope ratios as tracers in marine food webs: an update In: Michener RH, Lajtha K, editors. Stable isotopes in ecology and environmental science. 2nd ed Malden (MA): Blackwell Publishing; 2007 p. 238–82.

[36] McCutchan JH Jr, Lewis WM Jr, Kendall C, McGrath CC. Variation in trophic shift for stable isotope ratios of carbon, nitrogen, and sulfur. Oikos. 2003;102:378–90.

[37] Kelly FJ. Stable isotopes of carbon and nitrogen in the study of avian and mammalian trophic ecology. Can J Zool. 2000;78:1–27.

[38] Newsome SD, Clementz MT, Koch PL. Using stable isotope biogeochemistry to study marine mammal ecology. Mar Mamm Sci. 2010;26(3):509–72.

[39] McConnaughey T, McRoy CP. Food-web structure and the fractionation of carbon isotopes in the Bering Sea. Mar Biol. 1979;53(3):257–62.

[40] France RL. Carbon-13 enrichment in benthic compared to planktonic algae: foodweb implications. Mar Ecol Prog Ser. 1995;124:307–12.

[41] Graham BS, Koch PL, Newsome SD, McMahon KW, Aurioles D. Using isoscapes to trace the movements and foraging behavior of top predators in oceanic ecosystems In: West JB, Bowen GJ, Dawson TE, Tu KP, editors. Isoscapes: understanding movement, pattern, and process on Earth through isotope mapping. New York: Springer; 2010 p. 299–318.

[42] Hobson KA. Stable isotopes and the determination of avian migratory connectivity and seasonal interactions. Auk. 2005;122:1037–48.

[43] Kirsch PE, Iverson SJ, Bowen WD. Effect of diet on body composition and blubber fatty acids in captive harp seals (*Phoca groenlandica*). Physiol Biochem Zool. 2000;73:45–59. 10685906 10.1086/316723

[44] Hobson KA, Clarke RG. Turnover of 14C in cellular and plasma fractions of blood: implications for nondestructive sampling in avian dietary studies. Auk. 1993;110:638–41.

[45] Hildebrand GV, Farley SD, Robbins CT, Hanley TA, Titus K, Servheen C. Use of stable isotopes to determine diets of living and extinct bears. Can J Zool. 1996;74:2080–8.

[46] Zhao L. Tracing amino acid metabolism of harbor seals (*Phoca vitulina*) using stable isotope techniques [dissertation]. Fairbanks, AK: University of Alaska Fairbanks; 2002.

[47] Zhao LY, Schell DM, Castellini MA. Dietary macronutrients influence ^13^C and ^15^N signatures of pinnipeds: captive feed studies with harbor seals (*Phoca vitulina*). Comp Biochem Physiol A Mol Integr Physiol. 2006;143:469–47. 16459116 10.1016/j.cbpa.2005.12.032

[48] Kurle CM. Interpreting temporal variation in omnivore foraging ecology via stable isotope modelling. Funct Ecol. 2009;23(4):733–44.

[49] Stabeno PJ, Schumacher JD, Salo SA, Hunt GAJ, Flint M. Physical environment around the Pribilof Islands In: Loughlin TR, Ohtani K, editors. Dynamics of the Bering Sea. Fairbanks (AK): University of Alaska Sea Grant; 1999 p. 193–215.

[50] Coachman LK. Circulation, water masses, and fluxes on the southeastern Bering Sea shelf. Cont Shelf Res. 1986;5:23–108.

[51] Schumacher JD, Stabeno PJ. The continental shelf of the Bering Sea In: Robinson AR, Brink KH, editors. The sea: the global coastal ocean regional studies and synthesis. Vol XI New York: John Wiley and Sons; 1998 p. 869–909.

[52] Wilke F, Kenyon KW. The food of the fur seal in the Bering Sea. J Fish Wildl Manag. 1957;21:237–8.

[53] Kajimura H. Opportunistic feeding of the northern fur seal, *Callorhinus ursinus*, in the eastern North Pacific Ocean and eastern Bering Sea. U.S. Dep. Commer, 1984 NMFS-SSRF-779.

[54] Antonelis GA, Sinclair EH, Ream RR, Robson BW. Inter-island variation in the diet of female northern fur seals (*Callorhinus ursinus*) in the Bering Sea. J Zool (1987). 1997;242:435–51.

[55] Call KA, Ream RR, Johnson DS, Sterling JT, Towell RG. Foraging route tactics and site fidelity of adult female northern fur seal (*Callorhinus ursinus*) around the Pribilof Islands. Deep-Sea Res Part 2 Top Stud Oceanogr. 2008;55:1883–96.

[56] Nordstrom CA, Battaile BC, Cotte´ C, Trites AW. Foraging habitats of lactating northern fur seals are structured by thermocline depths and submesoscale fronts in the eastern Bering Sea. Deep-Sea Res Part 2 Top Stud Oceanogr. 2013;88–89:78–96.

[57] Kuhn CE, Ream RR, Sterling JT, Thomason JR, Towell RG. Spatial segregation and the influence of habitat on the foraging behavior of northern fur seals (*Callorhinus ursinus*). Can J Zool. 2014;92(10):861–73.

[58] Kooyman GL, Gentry RL, Urquhart DL. Northern fur seal diving behavior; a new approach to its study. Sci Total Environ. 1976;193:411–2. 10.1126/science.935876 935876

[59] Goebel ME. Northern fur seal lactation, attendance and reproductive success in two years of contrasting oceanography [dissertation]. Santa Cruz (CA): UC Santa Cruz; 1991.

[60] Gentry RL, Holt JR. Equipment and techniques for handling northern fur seals. U.S. Department of Commerce, 1982 Contract No.: NMFS-SSRF-758

[61] Hobson KA, Gibbs HL, Gloutney ML. Preservation of blood and tissue samples for stable-carbon and stable-nitrogen analysis. Can J Zool. 1997;75:1720–3.

[62] Freitas C, Lydersen C, Fedak MA, Kovacs KM. A simple new algorithm for filtering marine mammal Argos locations. Mar Mamm Sci. 2008;24(2):315–25.

[63] Johnson D, London J, Lea MA, Durban J. Continuous-time random walk model for animal telemetry data. Ecology. 2008;89:1208–15. 18543615 10.1890/07-1032.1

[64] Baylis AMM, Page B, Goldsworthy SD. Effect of seasonal changes in upwelling activity on the foraging locations of a wide-ranging central-place forager, the New Zealand fur seal. Can J Zool. 2008;86:774–89.

[65] Kuhn CE. The influence of subsurface thermal structure on the diving behavior of northern fur seals (*Callorhinus ursinus*) during the breeding season. Mar Biol. 2011;158:649–63.

[66] Team RDC. R: A language and environment for statistical computing. ISBN 3-900051-07-0. http://www.R-project.org. Vienna, Austria: R Foundation for Statistical Computing, 2006

[67] Bates D, Maechler M, Bolker BM, Walker S. lme4: Linear mixed-effects models using Eigen and S4. Journal of Statistical Software. 2014 25400517

[68] Iverson SJ, Frost KJ, Lang S. Fat content and fatty acid composition of forage fish and invertebrates in Prince William Sound, Alaska: factors contributing to among and within species variability. Mar Ecol Prog Ser. 2002;241:161–81.

[69] Aitchison J. Principal component analysis of compositional data. Biometrika. 1983;70:57–65.

[70] Ludwig JA, Reynolds JF. Statistical ecology: a primer on methods and computing. New York: John Wiley and Sons; 1988.

[71] Ward JH. Hierarchical grouping to optimize an objective function. J Am Stat Assoc. 1963;58:236–44.

[72] Hobson KA. Tracing origins and migration of wildlife using stable isotopes: a review. Oecologia. 1999;120:314–26. 28308009 10.1007/s004420050865

[73] Sullivan JC, Buscetta KJ, Michener RH, Whitaker JO, Finerty JR, Kunz TH. Models developed from C and N of skin tissue indicate non-specific habitat use by the big brown bat (*Eptesicus fuscus*). Ecoscience. 2006;13(1):11–22.

[74] Loughlin TR, Bengtson JL, Merrick RL. Characteristics of feeding trips of female northern fur seals. Can J Zool. 1987;65(8):2079–84.

[75] Kuhn CE, Baker JD, Towell RG, Ream RR. Evidence of localized resource depletion following a natural colonization event by a large marine predator. J Anim Ecol. 2014;83:1169–77. 24450364 10.1111/1365-2656.12202

[76] Sobolevskii EI, Sokolovskaya TG. New data on the biology of the smoothtongue, *Leuroglossus schmidti* (Bathylagidae), in the Northwestern Pacific. J Ichthyol. 1994;34(3):20–7.

[77] Kubodera T, Jefferts K. Distribution and abundance of the early life stages of squid, primarily Gonatidae (Cephalopoda, Oegopsida), in the northern North Pacific (parts I and II). Bull Natl Mus Nat Sci Ser A Zool. 1984;10:165–93.

[78] Robards MD, Wilson MF, Armstrong RH, Piatt JF. Sand lance: a review of biology and predator relations and annotated bibliography PNW-RP-521. Portland, OR: U.S Fish and Wildlife Service; 1999.

[79] Waite JN, Trumble SJ, Burkanov VN, Andrews RD. Resource partitioning by sympatric Steller sea lions and northern fur seals as revealed by biochemical dietary analyses and satellite telemetry. J Exp Mar Bio Ecol. 2012;416–417:41–54.

[80] Burton RK, Koch PL. Isotopic tracking of foraging and long distance migration in northeastern Pacific pinnipeds. Oecologia. 1999;119:578–85. 28307717 10.1007/s004420050822

[81] Burton RF, Snodgrass JJ, Gifford-Gonzalez D, Guilderson TP, Brown T, Koch PL. Holocene changes in the ecology of northern fur seals: insights from stable isotopes and archaeofauna. Oecologia. 2001;128:107–15. 28547080 10.1007/s004420100631

[82] Zeppelin TK, Orr AJ. Stable isotope and scat analyses indicate diet and habitat partitioning in northern fur seals *Callorhinus ursinus* across the eastern Pacific. Mar Ecol Prog Ser. 2010;409:241–53.

[83] Kurle CM. Stable-isotope ratios of blood components from captive northern fur seals (*Callorhinus ursinus*) and their diet: applications for studying the foraging ecology of wild otariids. Can J Zool. 2002;80:902–9.

[84] Lowther AD, Goldsworthy SD. Detecting alternate foraging ecotypes in Australian sea lion (*Neophoca cinera*) colonies using stable isotope analysis. Mar Mamm Sci. 2010;27(3):567–86.

[85] Davenport SR, Bax NJ. A trophic study of a marine ecosystem off southeast Australia using stable isotopes of carbon and nitrogen. Can J Fish Aquat Sci. 2002;59:514–30.

[86] Benoit-Bird KJ, Battaile BC, Nordstrom CA, Trites AW. Foraging behavior of northern fur seals closely matches the hierarchical patch scales of prey. Mar Ecol Prog Ser. 2013;479:283–302.

[87] Hunsicker ME, Essington TE, Aydin KY, Ishida B. Predatory role of the commander squid *Berryteuthis magister* in the eastern Bering Sea: insights from stable isotopes and food habits. Mar Ecol Prog Ser. 2010;415:91–108.

[88] Kurle CM, Sinclair EH, Edwards AE, Gudmundson CJ. Temporal and spatial variation in the d15N and d13C values of fish and squid from Alaskan waters. Mar Biol. 2011;158:2389–404.

[89] Gorbatenko KM, Kiyashko SI, Lazhentsev AY, Nadtochii VA, Savin AB. Benthic-pelagic trophic interactions within the fish assemblage in the western Bering Sea shelf area according to stomach content analysis and rations of C and N stable isotopes. Russ J Mar Biol. 2008;34(7):497–506.

[90] Hopkins JBI, Ferguson JM. Estimating the diets of animals using stable isotopes and a comprehensive Bayesian mixing model. PLoS ONE. 2012;7(1):10.1371/annotation/d222580b-4f36-4403-bb1f-cfd449a5ed74 PMC3250396 22235246

[91] Springer AM, Ream RR, Iverson SJ. Seasonal foraging strategies and consequences for northern fur seals at colonies with opposite population trends—Year 2. 2008 North Pacific Research Board Project 524 Final Report.

[92] Towell RG, Ream RR, Sterling JT, Williams M, Bengtson JL. Population assessment of northern fur seals on the Pribilof Islands, Alaska, 2010–2011 In: Testa JW, editor. Fur seal investigations, 2010–2011: U.S. Dep. Commer NOAA Tech. Memo. NMFS-AFSC-241; 2012 p. 8–25. 10.1177/0300985812465323

